# Patellar instability: current approach

**DOI:** 10.1530/EOR-2025-0051

**Published:** 2025-06-02

**Authors:** David H Dejour, David Mazy, Tomas Pineda, Nicolas Cance, Michael J Dan, Edoardo Giovannetti de Sanctis

**Affiliations:** ^1^ Lyon-Ortho-Clinic, Lyon, France; ^2^ CHU Sainte-Justine, Montréal, Canada; ^3^ Hospital el Carmen, Santiago, Chile; ^4^ Orthopaedics Surgery and Sports Medicine Department, FIFA Medical Center of Excellence, Croix-Rousse Hospital, Lyon North University Hospital, Lyon, France; ^5^ Surgical and Orthopaedic Research Laboratory, Prince of Wales Clinical School University of New South Wales, Sydney, Australia; ^6^ IULS-Institut Universitaire Locomoteur et Sports, Pasteur 2 Hospital, CHU, Nice, France

**Keywords:** patellar instability, medial patellofemoral ligament, patella alta, tibial tubercle osteotomy, tibial tubercle–trochlear groove, trochlear dysplasia, trochleoplasty

## Abstract

- Patellar dislocations present predominantly during adolescence, with a higher incidence observed among female patients.
- Patellofemoral joint stability depends critically on both osseous anatomy and soft tissue structures.
- Patellofemoral pathology can be classified into three major groups: objective patellar instability OPI, potential patellar instability and painful patellar syndrome.
- Three primary risk factors predispose individuals to patellar dislocation: trochlear dysplasia, patella alta and increased tibial tuberosity-trochlear groove (TT–TG) distance.
- Three secondary risk factors should be considered: femoral and tibial rotational abnormalities and valgus deformity.
- MRI has become the imaging modality of choice, enabling precise quantification of OPI risk factors in a single imaging examination.
- The ‘menu à la carte’ approach guides the treatment of OPI by addressing the most relevant anatomical risk factors for each patient using statistical thresholds.

## Introduction

Patellofemoral instability represents one of the most challenging clinical entities encountered by orthopaedic surgeons. Clinical evaluations typically yield unspecific findings, necessitating precise and advanced imaging modalities, including both plain radiographs and cross-sectional imaging, interpreted by clinicians possessing a thorough understanding of the relevant anatomical and pathological criteria (1). Thus, establishing an accurate diagnosis and defining an effective treatment algorithm can appear daunting; however, a systematic and structured approach greatly facilitates optimal patient management (1).

The present article aims to highlight the assessment and management strategies for patellar instability, including patient history, physical examination, imaging to rate risk factors and treatment algorithm.

## Definition and epidemiology of patellar instability

Typically, patellar dislocations present predominantly during adolescence, with a higher incidence observed among female patients. Reported incidence rates vary from approximately 5.8 to 29 per 100,000 individuals aged between 10 and 17 years (2). Patients affected often experience significant psychological impacts (3).

Patellofemoral (PF) joint stability depends critically on both osseous anatomy and soft tissue structures, directly influencing the functional integrity of the knee extensor mechanism. Anatomically, the lateral facet of the femoral trochlea exhibits greater prominence and height compared to its medial counterpart, mirroring the congruent shape of the patellar articular surface. This lateral trochlear prominence is crucial for resisting lateralizing forces acting upon the patella. In addition, the lateral retinaculum demonstrates increased strength and width relative to the medial retinaculum. Consequently, any imbalance favouring lateral forces, often arising from anatomical abnormalities, predisposes the patella to dislocation.

### Classification of patellofemoral pathology

Patellofemoral pathology can be classified into three major groups. H Dejour and the Lyon school introduced this classification (4); i) objective patellar instability (OPI): defined by at least one documented episode of lateral patellar dislocation coupled with an identifiable anatomical risk factor. Lateral patellar dislocation occurring during knee extension with a non-contact trauma is the most common presentation. Documented objective dislocation events diagnosed by a healthcare professional are necessary as subjective reports of instability, especially among adolescents, frequently confuse subluxations or quadriceps inhibition leading to reflex instability symptoms with actual dislocation. It is precisely this first category of patients that constitutes the main focus of the present article. Within the OPI/OPD group, further differentiation includes recurrent (more than three documented episodes), habitual (dislocations occurring consistently in early knee flexion) and permanent (persistent patellar displacement throughout the entire range of motion) subtypes. ii) Potential patellar instability/dislocation (PPI/PPD): characterized by the presence of anatomical risk factors associated with knee discomfort, but without any documented episodes of complete patellar dislocation. iii) Painful patellar syndrome (PPS): patients within this category present with knee pain without detectable anatomical risk factors or documented subluxation/dislocation events. It is important to note that many cases classified within PPS may not present patellar instability.

It is therefore essential to differentiate between instability and pain by questioning the patient appropriately. Pure traumatic dislocations, usually by a contact trauma, characterized by normal anatomical findings and notable difficulty in patellar relocation post-injury, are extremely rare and excluded from this classification.

### Specific genetic considerations

In addition, clinicians must be aware of specific genetic disorders predisposing patients to patellar instability. Among these conditions is the ‘nail-patella’ syndrome, a rare multisystemic disorder characterized, among other features, by a markedly hypoplastic or absent patella and fingernail dysplasia (5). The ‘small patella’ syndrome, although a distinct bony dysplasia, is also defined by patellar hypoplasia or aplasia (6). Consequently, the identification of patella hypo- or aplasia should prompt further investigation for an underlying genetic condition and referral for genetic consultation.

### Risk factors

A comprehensive understanding of anatomical risk factors associated with OPI is crucial for determining the optimal treatment pathway for each patient. Originally described by H. Dejour, four primary anatomical anomalies predispose individuals to patellar dislocation: trochlear dysplasia, patella alta, increased tibial tuberosity-trochlear groove (TT–TG) distance and patellar tilt (4). Over the past decade, these factors have been extensively validated, although the patellar tilt is now considered more as a consequence rather than an independent risk factor. This leaves three major risk factors (Table 1). 

**Table 1:** MRI assessment of risk factors: measures, imaging planes and thresholds.

Risk factor/measures	Planes	Thresholds
Trochlear dysplasia		
Spur	Sagittal	5 mm
CTO	Axial	Positive
TGA	Coronal	>11°
Patella alta		
PHI	Sagittal	1.16
SPE	Sagittal	0.38
TT–TG distance	Axial	14 mm

CTO, cranial trochlear orientation; FMA, femoral mechanical axis; PHI, patellar high index; SPE, sagittal patellofemoral engagement; TGA, trochlear groove axis; TT–TG, tibial tuberosity-trochlear groove.

#### Trochlear dysplasia

Trochlear dysplasia, characterized by a shallow or flattened femoral trochlear groove often accompanied by a supratrochlear prominence, is recognized as the primary anatomical risk factor, present in up to 96% of patients with OPI (4). The shape of the trochlea allows for greater lateral translation of the patella, thus promoting OPI. Facing the trochlear, the patella may be dysplastic and contributes to an increased tilt or maltracking.

#### Patella alta

Patella alta, defined as an abnormally elevated patellar position relative to the trochlea, constitutes the second primary risk factor capable of independently causing instability (7). This delays patellar engagement in early knee flexion, resulting in a prolonged free-floating patella, increasing the likelihood of OPI. In addition, Patella alta increases quadriceps force vectors and reduces patellofemoral contact area at early flexion angles, predisposing patients to cartilage degeneration and anterior knee pain.

#### Laterally directed force of the extensor mechanism: TT–TG distance

An excessive TT–TG distance represents the lateral displacement of the tibial tuberosity relative to the trochlear groove (9). This represents a method for evaluating the axial alignment of the extensor mechanism. Unlike trochlear dysplasia and patella alta, increased TT–TG distance alone rarely causes patellar dislocation, but contributes significantly when combined with trochlear dysplasia (1).

#### Other factors

Other, secondary risk factors, such as excessive femoral anteversion, external tibial rotation and valgus alignment, should also be systematically evaluated during clinical examination. If any suspicion arises, specific imaging studies should be performed to confirm and quantify these abnormalities (8).

In addition to osseous factors, soft tissue must also be considered. Patellofemoral joint stability depends on both passive and active stabilizing components. The medial patellofemoral ligament (MPFL) acts as the primary static stabilizer, providing up to 60% of the resistance against lateral patellar displacement (9). Importantly, a complete dislocation of the patella inevitably results in an MPFL rupture (10). In addition, the medial patellotibial ligament (MPTL) significantly contributes to patellar stability, especially in flexion, and is often involved in patellar avulsion injuries (11). On the lateral aspect, chronic instability and patellar malalignment commonly result in thickening and retraction of the lateral retinaculum. The dynamic stabilizer is the vastus medialis oblique muscle, which is frequently found hypoplastic or insufficient in OPI.

## Evaluation and assessment

### Physical examination

Clinical evaluation is essential for diagnosing patellar instability, as it provides key insights into both dynamic and static abnormalities influencing patellar tracking. Common patient-reported symptoms include anterior knee pain, a sensation of knee instability, episodes of locking and intermittent catching.

Specific clinical tests play an essential role in guiding the diagnosis and identifying potential anatomical risk factors for patellar instability.

Among these tests, the apprehension test is essential. This test involves gently applying lateral force to the patella, which induces anxiety or protective muscular resistance in patients who have experienced lateral patellar dislocation. A positive apprehension test strongly supports the presence of patellar instability.

The patellar tilt test, conducted with the knee fully extended, quantifies lateral inclination of the patella and helps to identify excessive lateral retinacular tightness or imbalance.

The medial tilt test further assesses the flexibility and tension of lateral soft tissue structures by evaluating whether the lateral patellar tilt can be passively corrected.

The quadrant test, performed in extension and flexion, assesses the competence and integrity of the MPFL by quantifying medial and lateral patella mobility according to quadrants of patellar width.

Patellar tracking is best evaluated with the patient seated at the edge of the examination table, actively or passively flexing and extending the knee. Maltracking describes dynamic malalignment of the patella within the trochlear groove during range of motion. A specific subtype of maltracking, the J-sign, occurs when the patella disengages laterally from the trochlea during terminal knee extension, tracing a characteristic ‘J-shaped’ path. This sign is often indicative of patella alta or a shallow, convex trochlea.

Furthermore, clinical examination must systematically include assessment for rotational alignment, notably femoral anteversion and external tibial rotation. Any clinical suspicion of rotational abnormalities or valgus alignment should prompt a precise evaluation through advanced imaging techniques to accurately quantify the deformities and facilitate appropriate therapeutic planning.

### Imaging screening

**Figure 1:**
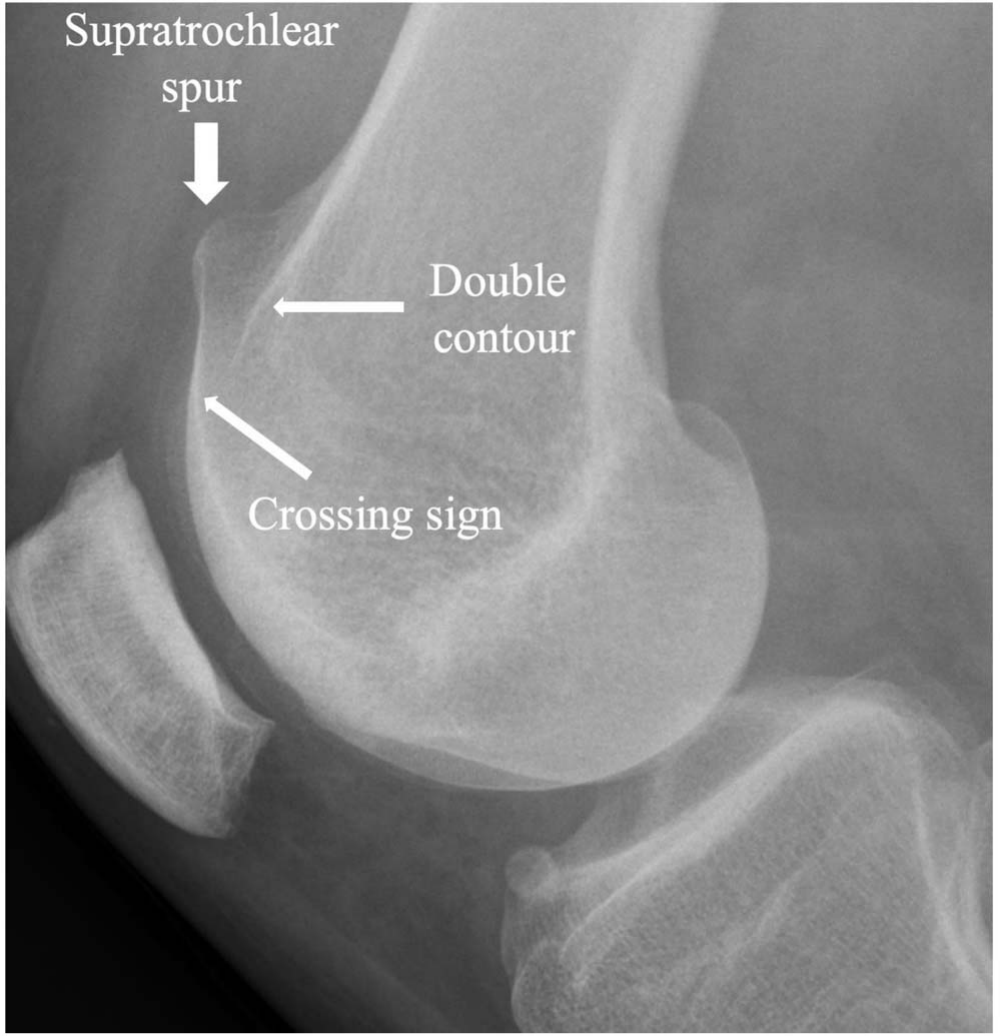
Three pillars of trochlear dysplasia visible on X-rays.

Although clinical examination remains essential and informative, diagnostic imaging allows precise quantification of risk factors, guiding appropriate therapeutic decision-making. Historically, lateral radiographs were initially employed to assess the three radiographic pillars of the trochlear dysplasia, such as the supratrochlear spur, crossing sign and double contour (4) (Fig. 1). In addition, patellar height evaluation was routinely performed using the Caton–Deschamps index (CDI), now established by the International Patellofemoral Study Group as the recommended method (12). Then, computed tomography (CT) improved classification of trochlear dysplasia and enabled precise quantification of the TT–TG distance, thus allowing assessment of the three major anatomical risk factors for OPI (1, 13).

**Figure 2:**
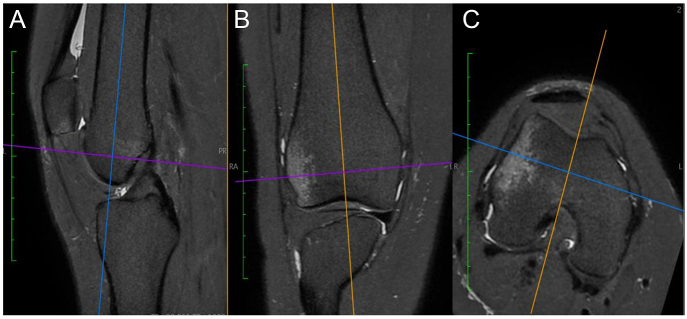
Multiplanar reformation mode enables reorientation of slices planes according to the femoral anatomical axes for sagittal (A) and coronal (B) images and to the posterior bicondylar line for the axial (C) images.

However, limitations in reproducibility and interpretation variability associated with radiographic and CT-based measurements have progressively shifted diagnostic preference towards magnetic resonance imaging (MRI). MRI has become the imaging modality of choice, enabling precise quantification of OPI risk factor in a single imaging examination. In addition, MRI allows reliable detection of associated lesions, such as osteochondral loose fragments, cartilage injuries or other concomitant lesions. It is mandatory to have the native acquisition (FOV, cube or space) and use the multiplanar reformation (MPR) to align the condyles and have a standardized MRI, which allows quantification assessment of trochlear dysplasia, patella alta and TT–TG distance, significantly refining the accuracy and reliability of preoperative evaluations (14) (Fig. 2). The value of the MRI is to have in one imaging the full screening of the PF joint, with statistical thresholds for trochlear dysplasia, patella alta and TTTG available for any surgeons in any orthopaedic institution over the world.

#### Trochlear dysplasia

MRI axial slices allow measurement of the sulcus angle, the lateral trochlear inclination (LTI) and assessment of the trochlear morphology (15).

**Figure 3:**
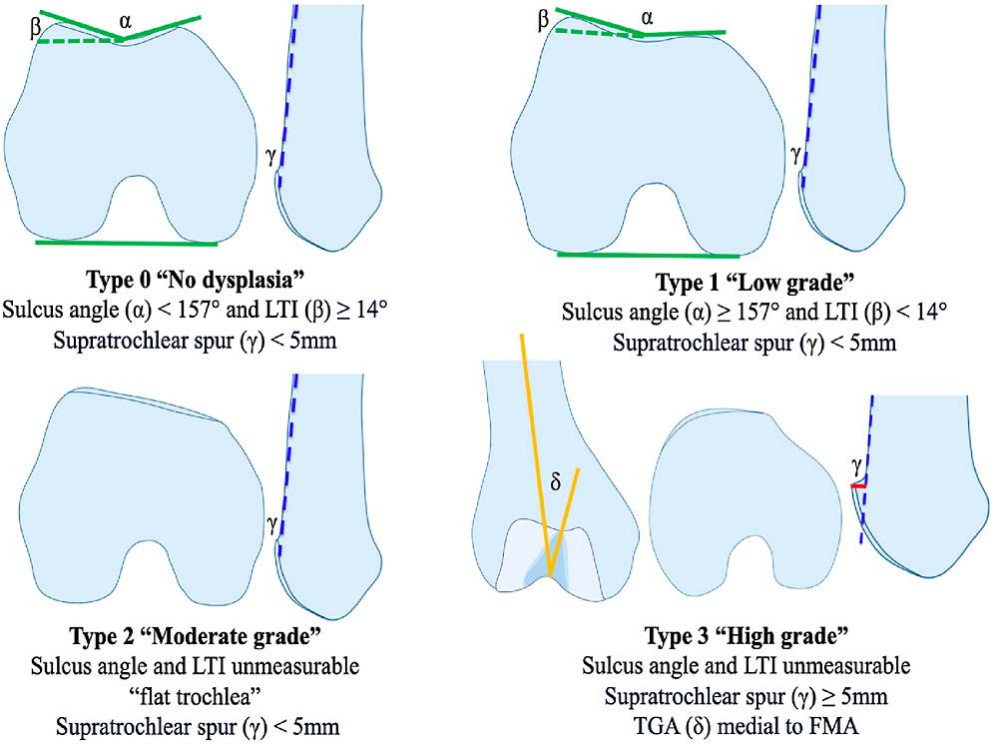
The quantitative MRI Dejour classification. LTI, lateral trochlear inclination; TGA, trochlear groove angle.

Trochlear dysplasia can be thoroughly characterized in all three spatial planes, enabling precise classification exclusively through MRI-based measurements (15) (Fig. 3).

Cranial trochlear orientation (CTO), measurable in axial views, further enhances diagnostic accuracy; a positive CTO, indicating a laterally oriented trochlea relative to the posterior bicondylar line, indicates high-grade dysplasia (16). Sagittal slices enable precise quantification of the supratrochlear spur height or bump (15). Assessment of the trochlear groove angle (TGA) on coronal slices identifies medial orientation relative to the femoral mechanical axis as another high-grade dysplasia indicator (17).

The MRI V.3 trochlear classification (ref) gives a linear progression from none dysplastic to high-grade trochlear dysplasia and therefore helps for planning the surgical.

#### Patella alta

**Figure 4:**
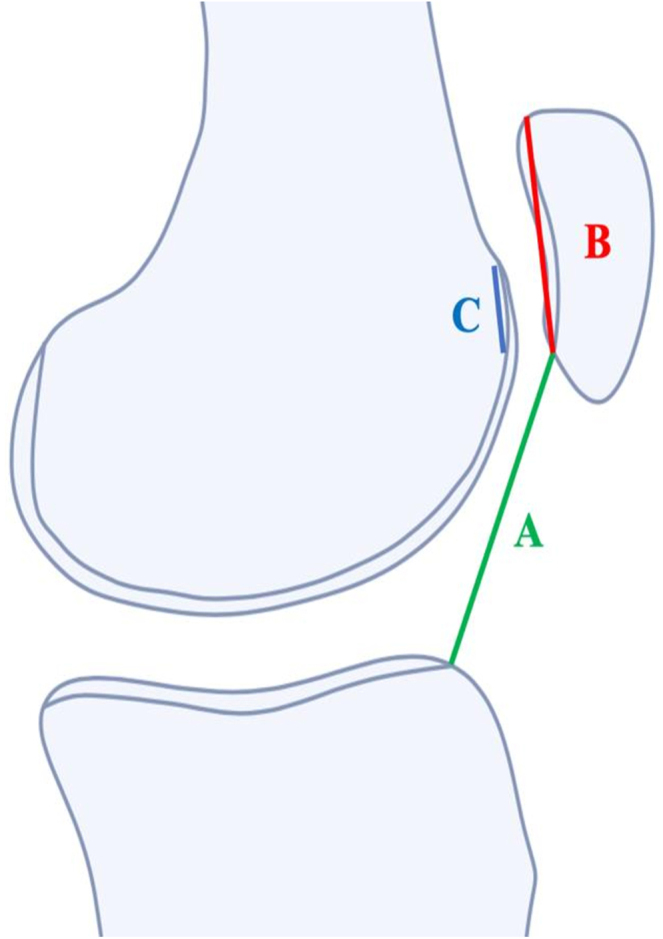
CDI (A/B) and SPE index (C/B).

MRI sagittal slices effectively quantify patellar height index (PHI), which corresponds to the ratio between the patellar cartilage lengh and the distance from the anterior part of the tibial plateau to the lower part of the patella. The measurement is done by the superimposition of the two cuts one passing through the central part of the patella and the second in the anterior cruciate ligament MRI slice, primarily using the CDI method with different values due to the position of the knee in the MRI coil. Beyond the PHI, the sagittal patellofemoral engagement (SPE) index has become increasingly valuable (18). This index, calculated as the ratio of the patellar articular cartilage length to the central trochlear cartilage length, serves as a supplementary measure to identify cases of insufficient patellofemoral engagement not detectable by standard CDI measurements alone (Fig. 4). The position of the patella should be related to the trochlea; therefore the simple evaluation of the patella height using CDI is not sufficient. The PHI and the SPE are two linked measurements and define the patella norma and the patella alta.

#### TT–TG distance

MRI provides precise measurement of the TT–TG distance using chondral reference points within the trochlear groove. Slices extending distal to the tibial tubercle insertion are essential to ensure accurate calculation. The threshold differs from the CT scan value; the threshold value is 14 mm while the CT scan value was 20 mm, these differences are related to the taken landmarks, tendon and cartilage for MRI and bone for CT scan.

## Treatment algorithm: '*menu à la carte*'

**Figure 5:**
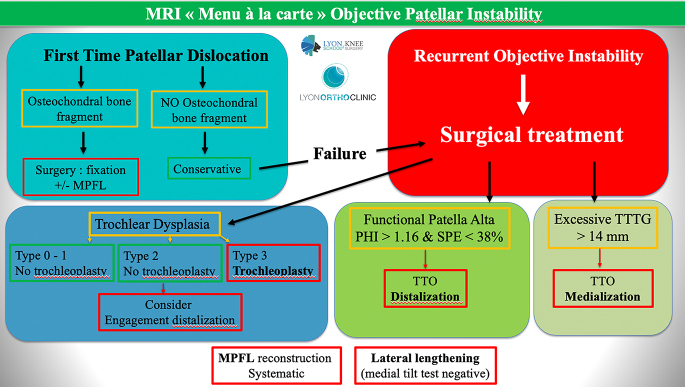
The updated treatment algorithm for patellar instability. MPFL, medial patellofemoral ligament; TT, tibial tuberosity; PHI, patellar high index; SPE, sagittal patellofemoral engagement; TT–TG, tibial tubercle-trochlear groove.

The ‘*menu à la carte*’ approach, initially proposed by the Lyon School of Knee Surgery in 1987, has guided the treatment of patellar instability by addressing one by one the most relevant anatomical risk factors for each patient. Over time, this strategy has evolved with advancements in imaging and classification systems, leading to this therapeutic algorithm’s most recent MRI-based adaptation (1, 15) (Fig. 5).

The MPFL acts as a static restraint to lateral translation of the patella. During acute lateral patellar dislocations, the MPFL is ruptured >90% of the time, with almost 100% rupture occurring in repeat dislocations (19). Therefore, the surgical planning includes systematically an MPFL reconstruction.

The choice of the procedures to be associated to the MPFL reconstructions is based on the evaluation of the trochlear dysplasia, the sagittal position of the patella and axial alignment.

The Dejour Classification V3.0 published in 2024 relying only on quantitative MRI measurements is based on three values: sulcus angle, the LTI and the central bump with cut-off values of respectively 150°, 14° and 5 mm (15).

- Type 0 (no dysplasia): sulcus angle <157 and LTI >14°.

- Type 1 (low-grade dysplasia): sulcus angle >157, LTI <14° and central bump <5 mm.

- Type 2 (moderate-grade dysplasia): sulcus angle/LTI unmeasurable and central bump <5 mm.

- Type 3 (high-grade dysplasia): sulcus angle >157 or unmeasurable, LTI <14° or unmeasurable and central bump >5 mm.

In Type 0 and 1, as there is no bump associated, any specific procedure to the trochlea should be added (e.g. trochleoplasty).

In Type 2, due to the altered shape (flatness/convexity), an associated distalization of the tibial tubercle might be considered, even in cases with normal patellar height, to improve patellofemoral congruence by allowing the patella to engage deeper within the trochlear groove. Trochlear procedures are not required in this type of dysplasia.

In type 3, a sulcus-deepening trochleoplasty (DT) should be considered to remove the bump and increase patellofemoral congruence.

The target goal in terms of sagittal position of the patella and axial alignment is to achieve, respectively, i) a postoperative patellar high index (PHI) <1.16 and a SPE >38% and ii) a TT–TG between 8 and 14 mm.

The medial tilt test is used to assess whether a lateral release should be associated.

### Nonoperative treatment

Nonoperative treatment remains the standard of care for first-time patellar dislocations, except in cases of osteochondral injury, loose bodies or associated injuries requiring surgical intervention. In select cases with a high risk of recurrence due to significant anatomical risk factors, early surgery may also be considered.

Early mobilization is recommended, as prolonged immobilization is unnecessary and may lead to muscle atrophy and joint stiffness. Range of motion exercises should begin as soon as tolerated, and weight-bearing should be allowed based on symptoms. A structured rehabilitation program focussing on quadriceps strengthening, neuromuscular control and proprioception is essential for restoring stability and reducing the risk of recurrence.

### Operative management: different techniques

#### MPFL reconstruction

The MPFL is the most important biomechanical restraint to lateral dislocation during early flexion. MPFL reconstruction (MPFLR) has demonstrated superior clinical outcomes and is therefore preferred over medial plication/repair of the soft tissues (20). Several techniques and graft options have been proposed, but none have shown clear superiority. Autografts are usually gracilis or semitendinosus hamstrings, or a quadriceps tendon turndown. Allografts are a good choice when available, offering low failure rates, particularly in patients with significant generalised ligamentous laxity.

As previously mentioned, in any patient with patellofemoral instability with previous dislocation, the use of the MPFL reconstruction is a cornerstone to the reconstruction algorithm. Isolated MPFLR is best suited for patients with Dejour V3 grade 1 trochlear dysplasia, without patella alta or an elevated tibial tuberosity–trochlear groove (TT–TG) distance. Otherwise, MPLFR is added systematically in addition to the other procedures from the menu à la carte approach at the end of the patella stabilisation procedure.

The senior author preferred surgical technique is described. The graft, typically the gracilis or semitendinosus, is harvested through either the tibial tubercle osteotomy (TTO) incision or a separate small incision over the pes anserinus insertion, as would be performed for a hamstring harvest in an anterior cruciate ligament reconstruction. A longitudinal incision is made over the medial border of the patella. The periosteum and medial retinaculum are raised subperiosteally, at which point the common medial ossicle that occurs with recurrent patella dislocations is excised. A 4.5 mm drill is used to create two converging drill holes through the anterior cortex of the patella, directed from anterior to posterior. This preserves an anterior cortical bridge. This is widened with a right angle artery forceps, before a shuttling suture is used to pass one end of the graft through the patella tunnel. The two ends of the graft are then sutured together in a whip stitch fashion, with the tails of the suture left long to allow the graft to shuttle through the 2nd and 3rd medial layer and then into the femoral tunnel. A 1.5 cm incision is made through the medial retinaculum, at the border of the vastus medialis obliquis (VMO), and the plane between the 2nd and 3rd medial layers is established towards the femoral insertion of the MPFL (21). The femoral insertion of the MPFLR is established using X-ray, with a small longitudinal incision made over the area of interest (22). A 2.4 mm guidewire is inserted, with proper lateral imaging to ensure accurate placement. A cannulated reamer larger than the doubled diameter of the graft, typically 6 mm, is then drilled. The guidewire is then used to pass a shuttling suture across the tunnel. The graft is passed deep to the medial retinaculum, facilitating VMO advancement, before being shuttled through the second and third medial layers. The graft is then passed into the femoral tunnel, the knee cycled through flexion, and then the graft is tensioned from the lateral side at 80 degrees of flexion. The knee is brought out into extension, two quadrants of lateral translation is confirmed with a firm end point. If not, the tension on the graft is reduced until this is the case. The knee is then flexed to 80 degrees of knee flexion again and a bioabsorbable screw, 1 mm larger than the tunnel size (typically 7 mm), is then used to fix the graft *in situ*. This tensioning technique helps avoiding issues related to over-tensioning the MPFLR, such as medial-sided pain and reduced knee flexion. The medial retinaculum is then closed with absorbable sutures.

#### Lateral release

The lateral release or lengthening should be performed always in association to other procedures and it is almost systematically associated with trochleoplasty. It is indicated in patients with a negative medial tilt. This procedure might be performed either arthroscopically or open.

When performed arthroscopically, an electrocautery is used to section the lateral tissue in such a way as to expose the distal extremity of the vastus lateralis proximally, extending to the lateral border of the patella distally.

When performed through open surgery (e.g. trochleoplasty), the subcutaneous plan must be dissected up to the external flare, while maintaining the initial incision. The section is then performed using an electrocautery, from proximal to distal.

#### Lower limb osteotomies

Valgus and rotational abnormalities (excessive femoral anteversion and external tibial torsion) of the lower limb can contribute to patellofemoral instability. Threshold values for these instability factors were not able to be established, and were therefore considered minor factors for instability (4).

Lower limb osteotomies should be considered when the three major localised factors for OPI are not identified in a patient with OPI.

For valgus deformity, a lateral distal femoral opening wedge osteotomy is preferred to a medial closing wedge osteotomy, as the medial plate fixation interferes with the MPFLR. The goal is correcting to a neutral mechanical coronal plan alignment utilising the reverse Miniaci method (23). In the skeletally immature patient, guided growth is recommended as a treatment option (20).

Rotational osteotomies should be considered when femoral anteversion or external tibial torsion exceeds to standard deviations above the mean. The aim is to correct these abnormalities to within normal limits. Unresolved femoral anteversion has been associated with poorer patient reported outcome scores in OPI (24). Femoral osteotomies can be performed at the subtrochanteric region, the femoral diaphysis or distal femur with plate or nail fixation. There is no established superiority for approach (25).

Tibial derotational osteotomies can be performed above (supratubercle), at the level or below the tibial tubercle (at the tibial shaft or at the supramalleolar region). The inclusion of the tibial tuberosity in the osteotomy is based on the TT–TG distance. Osteotomy above the tibial tubercle changes the TT–TG, as the tibial tubercle moves medially when the distal tibia is internally rotated. If the TT–TG is elevated, the tuberosity should be included in the rotation, and for every 1 degree of rotation, the TT–TG will be reduced by 0.6 mm (26).

#### TTO: medialization and distalization

The patella acts as the transmission component of the extensor mechanism, which ends distally on the anterior tibial tuberosity. Therefore, altering the position of this tibial tuberosity leads to a modification of the patella’s position. TTO is thus one of the key procedures in the treatment of patellofemoral instability and is part of the ‘menu à la carte’.

Two procedures are possible and often combined: i) distalization, which involves lowering the patellar height, and ii) medialization, which involves decreasing the TT–TG by medially shifting the distal insertion of the patellar tendon.

A patella alta (high patella) is defined on X-rays by a CDI >1.2. As discussed, it constitutes the second primary risk factor capable of independently causing instability, by positioning the patella outside the trochlear groove in extension. Actually, with the MRI evaluation, the decision making changed. The SPE is the factor which indicate a distalization if it is <38% and the PHI will determine the amount of distalization; an increased TT–TG >14 mm on MRI is an indication to combine medialization of the anterior tibial tubercle with surgical management, to recenter the patella in the trochlear groove by decreasing angle Q.

The technical execution of these two procedures is based on the same principles. A longitudinal incision is made over the anterior tibial tubercle, preferably along its medial edge (which also allows for a hamstring graft harvest via the same incision for MPFL reconstruction). Soft tissues are then carefully dissected to expose the tibial tuberosity and the insertion of the patellar tendon, which must be protected throughout the procedure to avoid damage to the extensor mechanism. A bone block is then marked: angles are prepared with a drill to prevent bone fracture, and the future screw holes are identified with a distance of 2 and 4 cm from the proximal edge of the bone block. The block should measure 6 cm in length and 1.5–2 cm in width.

It is important that, in cases requiring distalization, the bone block should measure 6 cm + the planned distalization to retain a sufficiently sized bone block. The preparation of the holes is then carried out using a 3.2 mm and a 4.5 mm drill and a countersink. The osteotomy is then performed using a thin and short oscillating saw, starting on the medial side. Once the bone block is harvested, it is necessary to traction it, checking the patella’s mobilization and the effect of the distalization, while freeing any soft tissue that might be obstructing. The cancellous (posterior) part of the bone block is then prepared with a gouge and the medial edge of the tibia to achieve optimal congruence when repositioning the bone fragment.

In the case of distalization, it is necessary to re-cut the bone block to remove the desired distalization on the distal part of the bone block. The bone block is then repositioned in the desired position, either lowered and/or medially shifted.

The planning of the bony procedure is crucial to achieve optimal correction of these two major risk factors for patellar instability. It is important to know that a pure distalization, due to tibial torsion, will result in an automatic medialization of 4 mm.

A square-tip clamp will be used to maintain the position while checking for perfect patellar tracking. If the patellar tracking is satisfactory, the bone block is fixed distally and then proximally using two 4.5 mm screws, without washers. The stability of the fixation is checked by applying a knee flexion of at least 90°.

#### Trochleoplasty

The trochleoplasty is a surgical correction of the trochlear groove shape and position, with the aim of improving the congruency of the articulating surfaces and preventing recurrent patellar dislocations (27).

**Figure 6:**
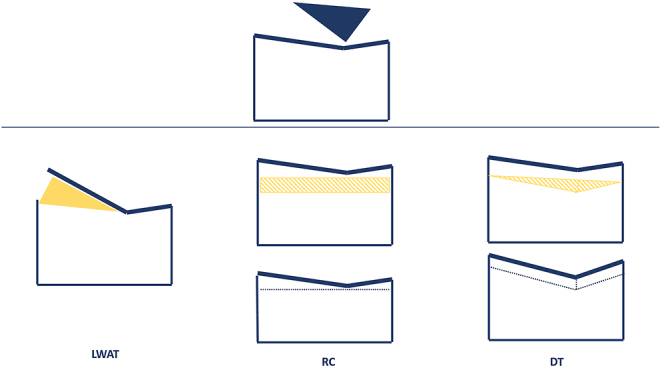
Different surgical techniques have been described to correct an abnormally shaped trochlea, which might be divided in three categories: the LWAT, RT and the DT.

Different surgical techniques have been described to correct an abnormally shaped trochlea, which might be divided in three categories: the lateral wedge augmentation trochleoplasty (LWAT), the recession trochleoplasty (RT) and the DT. Each technique has undergone many modifications since its first description (Fig. 6).

The goal of a trochleoplasty is to modify the shape of the groove, the prominence of the trochlea or both (Fig. 6). Unlike the LWAT and RT, which modify, respectively, the shape and the prominence, the aim of DT is to alter both features.

The LWAT was described by Albee in 1915 (28). This procedure’s aim is to elevate the lateral facet through a lateral opening-wedge osteotomy associated with bone grafting. This method is now considered to be erroneous and it has been gradually replaced, as it increases the patellofemoral joint reaction forces, with an increased risk of pain and osteoarthritis.

The RT described initially by Goutallier *et al.* was later modified by Beaufils *et al.* (29, 30). This procedure reduces the prominence without modifying the shape of the trochlear groove. A bone wedge is removed in order to allow the trochlea to settle into a deeper position.

The DT technique was described initially in 1978 by Masse *et al.* but then modified and standardized by H Dejour and later by D H Dejour (31, 32, 33). This procedure is known as the Lyon sulcus DT (thick-flap procedure or V-shaped DT) and is based on an osteotomy of both femoral condyles to create a V-shaped trochlear groove (34).

A different DT procedure known as ‘bereiter’ DT (or thin-flap procedure or U-shaped DT) was described, in which a thin osteochondral flap is raised from the trochlea and a bony sulcus is fashioned using burrs (35). This technique has been later described under arthroscopic control by Blønd and Schottle (36).

The principal difference between the two DT procedures lies in the preservation of cartilage and the thickness of the osteochondral flaps.

The trochleoplasty is considered a demanding procedure and it is frequently avoided due to a lack of familiarity. However, each patellofemoral physician should have it within the surgical armamentarium.

It has been shown that the trochleoplasty yields good clinical outcomes, whatever the technique used, in terms of outcome scores, patient satisfaction and postoperative dislocation rates, improving significantly the patients’ quality of life (27, 37).

Still controversial is whether the procedure might accelerate and increase the risk of the patellofemoral arthritis. Patients with trochlear dysplasia have an intrinsic higher rate of progression to patellofemoral osteoarthritis over time. The question is whether the degenerative wear in case of a high-grade trochlear dysplasia treated with a trochleoplasty is caused by the preexisting pathology or by the surgical procedure (27).

Here, we describe the most updated Lyon sulcus DT procedure (34).

The patient under sedation and regional anaesthesia is placed in a supine position. A 10 cm longitudinal midline skin incision with a transquadricipital tendon approach is performed. The supratrochlear synovial membrane is incised. The trochlear might then be prepared. The native groove, the medial and lateral facet limits are marked as dashed lines with a sterile marking pen. The new planned groove is then marked as continuous line. A strip of cortex is removed around the femorotrochlear osteochondral junction with an oscillating saw. The height of the removed bone wedge is equal to the prominence.

A special drill guide with a 5 mm off-set and a 4 mm high-speed burr are used to prepare the trochlear undersurface. Three ostechondral cuts along the marked lines are performed with a thin osteotome. The facets are rotated slightly to increase the sulcus angle and fixated with three BioComposite Labral SwiveLock Anchors 3.5 mm. The gaps between the facets are fulfilled with bone and the supratrochlear synovium is closed.

#### Patellar osteotomy

Patellofemoral stability is also determined by the patellar morphology (classified by Wiberg in 1941) and by the congruence of the trochlear and patellar articulating joint surfaces (38, 39).

The trochleoplasty alters the shape of the trochlear groove, leading in same case to a discrepancy between the morphology of the patellar articular facets with the modified trochlear groove, which may result in the persistence of patellar maltracking and elevated point pressures post-trochleoplasty. In these cases, a medial closing-wedge patellar osteotomy is indicated with the aim of recreating a medial and lateral patellar facet, forming an angle congruent with the reshaped trochlear groove (40).

## Conclusion

This summary reflects the experience gained during the past decades while treating the PF pathology. The key in this field is to quantify the anatomical abnormalities, using the threshold values, and then apply the ‘menu à la carte’ algorithm by correcting one by one the risk factors.

## ICMJE Statement of Interest

The authors declare that there is no conflict of interest that could be perceived as prejudicing the impartiality of the work reported.

## Funding Statement

The other authors, their immediate families and any research foundation with which they are affiliated have not received any financial payments or other benefits from any commercial entity related to the subject of this article.
